# Photophobotaxis of single‐celled and filamentous cyanobacteria

**DOI:** 10.1111/php.70051

**Published:** 2025-11-13

**Authors:** Maria Sinetova, Nina Spohrer, David Gabrielyan, Luke Lehmann, Julian Breinich, Dmitry Los, Tilman Lamparter

**Affiliations:** ^1^ Institute of Plant Physiology Moscow Russian Federation; ^2^ Josef Kölreuter Institut für Pflanzenwissenschaften JKIP Karlsruher Institut für Technologie Karlsruhe Germany

**Keywords:** cyanobacteria, light trap, movement, PixJ, random move model, type IV pili

## Abstract

Phototaxis refers to an organism's movement toward a light source, while photophobotaxis involves movement into illuminated regions. Although phototaxis in cyanobacteria has been widely studied, photophobotaxis has been investigated in only a few species. In this study, we examined photophobotaxis of 7 single‐celled and 11 filamentous cyanobacterial species, among them 3 Nostocales (filaments with heterocysts) and 5 Oscillatoriales and 1 Desertifiliales member. All single‐celled species and all Oscillatoriales/Desertifiliales exhibited photophobotaxis, whereas no evidence of photophobotaxis was found for the Nostocales and two other species. A pilus‐free mutant of *Synechocystis* sp. PCC 6803 did not display this behavior. The photosystem II inhibitor DCMU disrupted photophobotaxis in single‐celled and filamentous cyanobacteria at a concentration of 10 μM; only the filamentous *Phormidium lacuna* (*P. lacuna*) required 100 μM DCMU for inhibition. This points to PS II as a sensor of photophobotaxis. The widespread occurrence of photophobotaxis aligns with the universality of photosystems. Previous studies on spectral sensitivity and the cyanobacteriochrome PixJ in *P. lacuna* identified PixJ as a negative regulator of photophobotaxis. In pixJ mutants, light sensitivity was increased compared with the wild‐type. Dual‐wavelength experiments confirmed that yellow light induces PixJ to downregulate photophobotaxis. Our experiments also show that *P. lacuna* moves faster in darkness than in light and that a temporal change of light intensity from light to dark can induce a change of movement direction. Both findings support the light trap model which is based on random movement and a change of movement direction at the light–dark border.

## Graphical abstract

LED illumination from below induces central accumulation of cyanobacterial species.

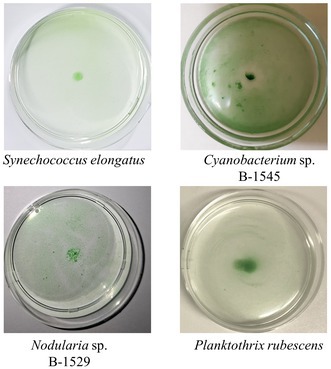

**Abbreviations**
DCMU: 3‐(3,4‐dichlorophenyl)‐1,1‐dimethylurea
LED: light emitting diode

## INTRODUCTION

Cyanobacteria are a group of bacteria that live on oxygen‐producing photosynthesis. Their organizational form is either single‐celled (e.g., Chroococcales), filamentous (e.g., Oscillatoriales), or filamentous with heterocysts (e.g., Nostocales).^1^ Most cyanobacteria can undergo gliding movements on surfaces, driven by type IV pili.^2^, ^3^ This movement is directed by light. Two kinds of directional movement can be distinguished: phototaxis^4^ and photophobotaxis.^3^, ^5^ During phototaxis, cells or filaments move in the direction of incident light, which is projected in parallel to the surface along which cyanobacteria glide. During photophobotaxis, a light beam comes perpendicular to the surface, either from above or from below, and the cyanobacteria become trapped in the illuminated area. Phototaxis has been studied on ca. 20 cyanobacterial species, with the focus on *Synechocystis* sp. PCC 6803.^6^, ^7^, ^8^ Photophobotaxis has been reported for the filamentous *Phormidium uncinatum* ^9^ and recently for the related *Phormidium lacuna*.^3^ In one experiment of a more recent publication, a photophobotactic effect was also described for *Synechocystis* sp. PCC 6803.^5^ Thus, comparatively little is known about photophobotaxis. Several photoreceptors have been discussed for phototaxis. PixJ, a cyanobacteriochrome consisting of one to five GAF domains in series and one methyl‐accepting chemotaxis sensor domain is somehow involved in phototaxis.^3^, ^10^, ^11^ Knockout mutants result in either a reversion of phototaxis or a loss of phototaxis. Photophobotaxis of *P. lacuna* was affected by the loss of PixJ by responding more sensitively to light. PixJ could play a role as a modulator of photophobotaxis, but the role of this protein is neither clear in phototaxis nor in photophobotaxis. Inhibitory action of DCMU on light direction sensing has been reported for several cyanobacteria including *P. lacuna*. If specific, these effects argue for a role of photosystem II as a light direction sensor. In the case of *P. lacuna* photophobotaxis, effective concentrations of DCMU were 100 μM or higher, questioning the specificity. Phototaxis of *Synechocystis* sp. PCC 6803 was unaffected by DCMU, but the movement of a *cph2* mutant toward blue was inhibited.^12^ Here, we tested 18 different species for photophobotaxis to get an overview of the universality of this light effect. We also performed more photophobotaxis experiments with *P. lacuna* for a better understanding of the mechanism.

## MATERIALS AND METHODS

Most species of the present study are from the collection of cyanobacteria of the Institute of Plant Physiology of the Russian Academy of Sciences. These species contain the term IPPAS in their names. The detailed species names are given in Table 1, together with an overview of the results. The strains were grown in ASP2, BG‐11, BG‐11‐N,^13^ Gromov 6‐N, Zarrouk, Zarrouk‐N,^14^ or f/2^15^ liquid medium. The specific growth medium is also indicated in Table 1. Typically, propagation occurred under shaking (60 rpm) and constant fluorescent light of 60 μmol m^−2^ s^−2^. For photophobotaxis, 8 mL cells with an OD_750 nm_ of ca. 0.2 were poured into Petri dishes of 5 cm diameter and placed on an LED holder with a single red LED (655 nm, 10 μmol m^−2^ s^−1^) in the center as described earlier.^3^ After an incubation time of 12 h to 7 days a picture of the Petri dish was taken. Usually, three or more repetitions were made; in some cases, additional experiments were performed as outlined in the Results section. For DCMU treatment, a 100 mM DCMU stock solution in ethanol was diluted into the medium to achieve final concentrations of 10 μM or 100 μM. The used concentration was based on preliminary experiments. In several cases, controls with 0.1% ethanol were made; these were comparable with the responses without ethanol. In almost all cases, dark controls were recorded. These controls show whether a pattern similar to a photophobotaxis response can form without light, which could contradict the interpretation.

**TABLE 1:** List of species used for photophobotaxis and quantification of results.

Species; medium; publication	Order	Photophobotaxis	DCMU	DCMU	Dark control
		(y/n;days;intensity)	10 μM	100 μM	
*Synechocystis* sp. PCC 6803; B; 1	Chroococcales	y; 2; 46 ± 10	i; 1 ± 2		3 ± 4
*Synechocystis* sp. PCC 6803; pili mutant; B	Chroococcales	n; 2; 3 ± 4	i; 3 ± 2		0 ± 2
*Synechococcus elongatus; B*	Synechococcales	y; 2; 39 ± 3	i; 2 ± 2		0 ± 1
*Geminocystis* sp. IPPAS B‐1530; B	Chroococcales	y; 3; 25 ± 9	i; 3 ± 3		2 ± 3
*Inacoccus* sp. IPPAS B‐1205; B	Chroococcales	y; 2; 51 ± 13	i; −1 ± 2		2 ± 1
*Cyanobacterium* sp. IPPAS B‐1545; Z	Chroococcales	y; 0.5; 84 ± 12			2 ± 2
*Cyanobacterium* sp. IPPAS B‐1200; Z	Chroococcales	y; 4; 59 ± 6	i; −3 ± 3		−1 ± 3
*Cyanobacterium* sp. IPPAS B‐2031; Z; 2	Chroococcales	y; 4; 61 ± 7			−3 ± 2
*Dolichospermum* sp. IPPAS B − 1213; G; 3	Nostocales	u; 6; 32 ± 1			
*Nodularia* sp. IPPAS‐1529; Z;8	Nostocales	u; 7; 37 ± 2			1 ± 2
*Desmonostoc* sp. IPPAS B‐1537; B	Nostocales	n; 2; −1 ± 2			−1 ± 1
*Halomicronema* sp. IPPAS B − 2022; Fx3; 9	Nodosileneales	n; 1; 1 ± 2			1 ± 2
*Toxifilum* sp. IPPAS B‐2086; A	Oculatellales	n; 7; 2 ± 0.4			−2 ± 4
*Desertifilum tharense* sp. IPPAS B‐1220; B; 10	Desertifilales	y; 3; 46 ± 8	i; −1 ± 1	i; 6 ± 4	2 ± 1
*Planktothrix rubescens PCC7821; B; 5*	Oscillatoriales	y; 2; 67 ± 16	i; 0 ± 3	i; 3 ± 2	
*Phormidium lacuna HE10DO; F; 6*	Oscillatoriales	y; 2; 127 ± 8		i; −1 ± 2	2 ± 3
*Sodalinema gerasimenkoi* sp. IPPAS B‐353;S; 7	Oscillatoriales	y; 0.7; 66 ± 9			−5 ± 4
*Oscillatoria lacuna; F*	Oscillatoriales	y; 2; 100 ± 23			4 ± 3
*Limnospira* sp. IPPAS B‐1526; Z; 8	Oscillatoriales	y; 4; 70 ± 12	i; −2 ± 3		−3 ± 3

*Note*: An abbreviation of the medium used is given after the species name in the first column; A stands for ASP2^16^; B for BG‐11 or BG‐11 without nitrogen^13^; Z for Zarrouk^14^; F for f/2^15^; G for Gromov 6‐N^16^; S for S medium.^17^ This entry is followed by a number which stands for the citation. 1^18^; 2^19^; 3^20^; 4^15^; 5^21^; 6^22^; 7^17^; 8^23^; 9^24^; 10^25^. Species without numbers are unpublished (see text). Column 3 shows the results of photophobotaxis (y = yes, n = no and u = unclear, see text) followed by the duration of irradiation in days and the mean pixel intensities ±SE of 3 or more experiments (see methods section). The i in the DCMU columns stands for inhibition. Empty field means the experiment was not performed.

Quantifications were made by the difference of the pixel intensity of a spot in the central area of the response and of a spot outside this area which is representative of the Petri dish. If no photophobotaxis was observed, a spot intensity in the central region was taken and a spot intensity of a nearby region was subtracted.

Time‐lapse studies were performed using infrared and red LED and a camera system as described before.^5^ For time lapse, pictures were usually stored in 1 min intervals. Other experiments were performed as described in the Results section.

## RESULTS

### Photophobotaxis of 18 cyanobacteria species

Photophobotaxis has rarely been investigated in cyanobacteria. Aside from our analyses of *Phormidium lacuna* and *Synechocystis PCC 6803* ^3^, ^5^ we are aware of results for only one other species, *Phormidium uncinatum*.^9^ While some relevant studies might have been overlooked, possibly conducted under different terms, we considered it important to gather additional information on other cyanobacterial species. Such investigations provide insights into the generality of the photophobotactic process.

The assay developed for *P. lacuna* ^3^, ^5^ is straightforward. It requires only a dark room, a single LED, a Petri dish, and a camera. The cyanobacterial culture is poured into the Petri dish, and the LED is positioned below, shining upward through the bottom of the dish for a defined period, while the rest of the cells or filaments remain in darkness. Photophobotaxis produces a characteristic pattern in which cells are enriched directly above the LED.

**FIGURE 1:**
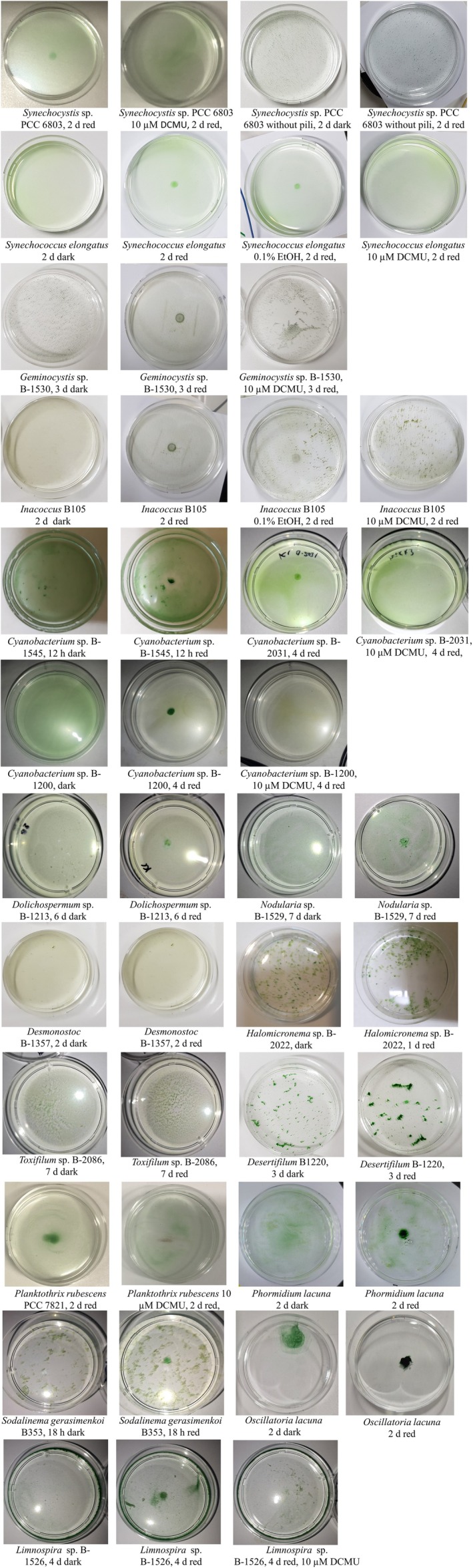
Photophobotaxis of cyanobacteria species in Petri dishes. Species are also listed in Table 1. Typical examples for most but not all experiments are shown. The “IPPAS” is removed from the species names; please refer to Table 1 and the text for complete names. Treatments are given under each panel.

Using this setup, we tested seven unicellular species from the orders Chroococcales and Synechococcales, three filamentous heterocystous species from the order Nostocales, five filamentous nonheterocystous species from the order Oscillatoriales, as well as members of the Oculatellales, Desertifilales, and Nodosilineales. Representative photographs for each species are shown in Figure 1. The list of species and the quantified results are given in Table 1. In almost all cases, we also captured images of control cultures kept in complete darkness for the same duration, to rule out nonlight‐related causes of cell aggregation at the center.

The typical irradiation period was 2 days or less, following the protocol of *P. lacuna*. If during this time a clear circular pattern above the LED appeared, we attributed this to photophobotaxis rather than photosynthetic growth, for several reasons: (i) The growth rate of the tested species is slower than that of the fast‐growing *Synechocystis* sp. PCC 6803, which shows a typical photophobotactic response. The pilus‐deficient mutant of *Synechocystis* does not show this response. (ii) Without photophobotaxis, cells or filaments generated by photosynthetic growth would move out of the irradiation zone, and the pattern would not remain stable. (iii) In several cases (e.g., *Cyanobacterium* sp. B‐1545), the area surrounding the LED exhibited a lower cell or filament density than the region at the periphery, indicating that cells or filaments moved into the illuminated area. (iv) For immobile cells or filaments, photosynthetic growth would produce an irregular distribution in the illuminated area, as observed for the pilus‐deficient mutant of *Synechocystis* sp. PCC 6803 and the Nostocales members *Dolichospermum* IPPAS B‐1213 and *Nodularia* sp. IPPAS B‐1529 (see below).

If no clear response was detected within 2 days in preliminary experiments, the duration of irradiation was extended to a maximum of 7 days. For most species, we still assume that photosynthetic growth contributed little or not at all to the observed accumulation, and that photophobotaxis was the dominant cause. In general, the above arguments (ii) (movement out of the zone without taxis) and (iv) (irregular pattern in immobile strains) still apply. Exceptions will be discussed below.

To further investigate the process, we tested the light responses of 10 species in the presence of DCMU (3‐(3,4‐dichlorophenyl)‐1,1‐dimethylurea) at concentrations of 10 μM or 100 μM, the concentration being dependent on preliminary experiments. DCMU is known to inhibit photophobotaxis, providing insights into the role of photosynthesis in this light direction process.^5^, ^8^ The inhibitory effects observed in our experiments align with the reported influence of photosynthesis on photophobotaxis.

### All single‐celled species except a pilus‐free mutant perform photophobotaxis

#### *Synechocystis* sp. PCC 6803

This single‐celled species, which is probably the most often used cyanobacteria research object worldwide, undergoes clear photophobotaxis. This has recently been published in an earlier article.^5^ Here, we repeated the experiments in the context of the present experiments and performed assays with the pilus‐defective mutant (Figure 1 and Table 1). For the wild‐type, we found again a clear response. We noted that shaking can result in a gathering of cells in the center of the Petri dish, thus mimicking photophobotaxis. Therefore, pictures were always taken with care without moving the plates during or after the irradiation. The photophobotaxis response was completely inhibited with 10 μM DCMU. The pilus‐free mutant^26^ did not undergo photophobotaxis (Figure 1 and Table 1), although we recognized a slight gathering at the edge of the illuminated area, which is hardly to be seen in the Figure 1 panel. This is probably indicative of photosynthetic growth as discussed above.

#### Synechococcus elongatus

This single‐celled species was collected at Lago di Como in Italy, a freshwater lake, brought into the lab and identified by an identical 16 S rRNA sequence as *Synechococcus elongatus*. Several strains of *Synechococcus elongatus* are used by different groups.^27^ The present strain was so far only used in practical courses but not in published studies. A photophobotaxis response was recognized after 2 days (Figure 1), and it further increased subsequently. About 10 μM DCMU inhibited the response completely (Figure 1 and Table 1).

#### *Geminocystis* sp. IPPAS B‐1530

Gathering of this single‐celled species in the light zone was observed after 4 hours, becoming more pronounced after 3 days, following the common pattern. The response was variable but in all cases clearly visible. No response was observed with 10 μM DCMU (Figure 1 and Table 1).

#### *Inacoccus* sp. IPPAS B‐1205

Already before the light treatment, the cells formed irregular aggregates in the Petri dish. In the light beam, a pattern according to the shape of the light beam could be recognized, that is, a part of the cells performed photophobotaxis. In some cases, the lighted area was empty and some cells accumulated at the edge (which we also consider photophobotaxis); in other cases, the illuminated area became filled with cells. With 10 μM DCMU, gathering in the light was completely inhibited or almost completely inhibited (Figure 1).

#### *Cyanobacterium* sp.

We investigated three strains of the genus “*Cyanobacterium*,” namely, *Cyanobacterium* sp. IPPAS B‐1545, *Cyanobacterium* sp. IPPAS B‐1200 and *Cyanobacterium* sp. IPPAS B‐2031. In all cases, we found a photophobotaxis response, although the duration of the light treatment to establish gathering in the light varied from 12 h to 4 days (Figure 1 and Table 1). The region around the central accumulated cells was often more translucent than the region at the periphery of the Petri dish, indicating that only cells close to the light could migrate to the light. Accumulation of cells in the light was tested in the presence of 10 μM DCMU for *Cyanobacterium* sp. IPPAS B‐1200; the response was inhibited (Figure 1 and Table 1). With *Cyanobacterium* sp. IPPAS B‐1545 and *Cyanobacterium* sp. IPPAS B‐1200, we followed the pattern after the light treatment and found that the spots became loose and floating. This shows that the cells are motile and a light trap is required to keep the cells together in the center; that is, this speaks against photosynthesis as an origin for the response.

### Light response for two out of three Nostocales members

The order Nostocales encompasses filamentous cyanobacteria with nitrogen‐fixing cells known as heterocysts. Vegetative filaments of *Nostocales* are generally considered immobile, with motility typically occurring only in hormogonia—shorter filaments that lack heterocysts.^28^ Surprisingly, we found a light response for two of the three Nostocales members tested (*Dolichospermum* sp. IPPAS B‐1213 and *Nodularia* sp. IPPAS B‐1529) (Figure 1 and Table 1). However, this response was detected after prolonged irradiation periods of 6–7 days. The filament suspensions were low (OD_750nm_ = 0.1) to minimize aggregation. For both species, the pattern in the center was irregular with several spots of higher filament densities within the illuminated area. Upon transfer to darkness, the spots remained stable for several days. This could indicate that the accumulation arose from photosynthetic growth of immobile filaments. The third Nostocales member, *Desmonostoc* sp. IPPAS B‐1537, showed no evidence of photophobotaxis after a light exposure of 2 days (Figure 1 and Table 1).

### No photophobotaxis for Nodosilenales and Oculatellales members

*Halomicronema* sp. IPPAS B‐2022 that belongs to the order Nodosileneales is probably (also) immobile, as the founding member of this genus is reported to be immobile.^29^ In accordance, our strain did not perform photophobotaxis (Figure 1 and Table 1). The founding member of the genus Toxifilum^30^ is reported to undergo twitching motility. In our case, no photophobotaxis response was observed for *Toxifilum* sp. IPPAS B‐2086.

### Desertifilales and Oscillatoriales perform photophobotaxis

#### *Desertifilum* sp. IPPAS B‐1220

Under experimental conditions the filaments of this *Desertifilum* species that belongs to Desertifilales, formed small aggregates. On this basis, a clear photophobotaxis was difficult to observe, but in the illuminated center area, a spot of filaments was seen in all three trials after a 3 days illumination period (Figure 1 and Table 1). We interpret these spots as a result of photophobotaxis, because of their flat appearance and a color that was slightly different from aggregates. Such spots were not found in the dark control (Figure 1) nor in the 10 μM DCMU or 100 μM DCMU samples (Table 1). Time‐lapse recordings (which were usually not performed in other cases) showed that on the plastic surface all filaments make gliding movements as other Oscillatoriales.

#### *Planktothrix rubescens* sp. PCC 7821

This Oscillatoriales species was obtained from the Pasteur culture collection of cyanobacteria (PCC) stock center. In our lab, it lost its originally red color and turned green. The photophobotaxis response was comparable with that of *P. lacuna*, but was inhibited with 10 μM DCMU (Figure 1 and Table 1). We observed here again that shaking can result in a gathering of filaments in the center of the plate (see also *Synechocystis* sp. PCC 6803 results above), thereby roughly mimicking photophobotaxis. This effect results probably from an arrangement of the extracellular matrix, together with a physical effect.

##### *Phormidium lacuna* HE10DO

*P. lacuna* (Oscillatoriales) has been analyzed in earlier work^3^, ^5^ and further experiments will be described below. To compare with the results of the other species here it is to be mentioned that the pixel intensity value was the highest one of all samples under investigation (Table 1). About 100 μM DCMU reduced the response completely (Figure 1 and Table 1). Note that the DCMU result is only given in Table 1 and not in Figure 1. In earlier work, 1000 μM DCMU resulted in a complete block of photophobotaxis, whereas the inhibition by 100 μM was incomplete.^5^ We also found that the spot disappeared after the cultures were brought from the LED light to darkness.

#### *Sodalimena gerasimenkoi* sp. IPPAS B353

This species is closely related to *P. lacuna*.^31^ Both have the same 16 S rRNA and PsbA sequences and 93% identical Phe‐tRNA ligase ß as examples. The response to light was similar to *P. lacuna* (example in Figure 1) but was not investigated for DCMU. Like in the other species above, the spot disappeared when the culture was brought from the LED to darkness.

#### Oscillatoria lacuna

This species was collected at the same rockpool environment from the North Sea coast of Helgoland Island as *P. lacuna*. Genome sequencing showed that it is a new species belonging to the genus *Oscillatoria*. We denominated it *Oscillatoria lacuna* to express its environmental origin (lacuna = pool). The filaments are larger and the speed of movement is slower as compared with *P. lacuna*. We performed nine standard experiments and six dark controls with *O. lacuna*. In all light‐treated samples, almost complete aggregation was observed already 1 day after the start of irradiation, as observed during brief inspections. At day 2, six out of nine aggregates were above the position of the LED or partially above that position (Figure 1), the three others were outside the center, that is, clearly not at the position of the light. Note that the quantification in Table 1 considers pixel intensities at the position of the LED, which were very variable due to the different positions of the aggregates. All dark controls formed aggregates as in Figure 1 that were significantly larger than those in the light. These were never in the center. The different aggregation between dark and light indicates that light must stimulate aggregation. This aggregation is probably not the result of a typical photophobotaxis, because not all aggregates were above the LED. When Petri dishes with aggregates from 2 days of illumination were kept for another 3 days with the LED at a peripheral position of the Petri dish, a typical, but weak, photophobotaxis pattern arose at this position, indicative of a regular photophobotaxis response (six repetitions). We also performed two experiments on agar plates. In both plates, all filaments were first arranged in a central line. An LED was placed at the periphery of the Petri dish. After 3 days, a minor fraction of filaments had migrated to that position. We therefore assume that *O. lacuna* can also undergo regular photophobotaxis.

In summary, we propose that *O. lacuna* can undergo photophobotaxis and light‐induced aggregation. The aggregation process might be initiated by regular photophobotaxis and proceed as a light‐independent process; otherwise, all aggregates would be found in the center of the Petri dish.

#### *Limnospira* sp. IPPAS B‐1526

The genus *Limnospira* is also named as *Spirulina* or *Arthrospira*. Preparations of this genus cultures are sold as food supplements which makes members of this genus interesting for applied investigations. *Limnospira* sp. IPPAS B‐1526 of the present study undergoes twitching motility and a clear photophobotaxis response. The effect was inhibited by 10 μM DCMU (Figure 1 and Table 1). When the culture was brought from the LED to darkness, the spot spread out and became formless, showing again that the filaments are motile and require a light trap for accumulation.

### Change of movement direction in red and dark

For more detailed understanding of photophobotaxis, we performed experiments with *P. lacuna* based on concepts that arose from earlier studies. The intention was to find out how light intensity changes are recognized.

In time‐lapse recordings, it was earlier found that filaments move continuously and change their direction randomly, whereas filaments that cross the light–dark border change their direction by ca. 180° and thereby remain in the light.^5^ These time‐lapse recordings were now repeated under various cell densities. In all cases, the same phenomenon was observed. For this publication, we selected a video in which the return of movement direction is most clearly seen (Video S1). In the video, accumulation in the light zone has already preceded.

We found that the movement speed of the filaments on the Petri dish plastic differs between red light and dark. In red light, the filaments moved ca. 50 μm min^−1^, in dark the speed was significantly higher at 130 μm min^−1^. The movement direction changed more often in red than in dark (Table 2). Slower movement and more frequent changes of movement direction in the light add up to the light trap effect.

**TABLE 2:** Movement of filaments and changes of movement direction recorded in the red area or in the dark (infrared) area.

	Red light	Dark (infrared)
Speed μm min^−1^	48 ± 3	130 ± 5
Direction changes min^−1^	0.65 ± 0.05	0.35 ± 0.02

**FIGURE 2:**
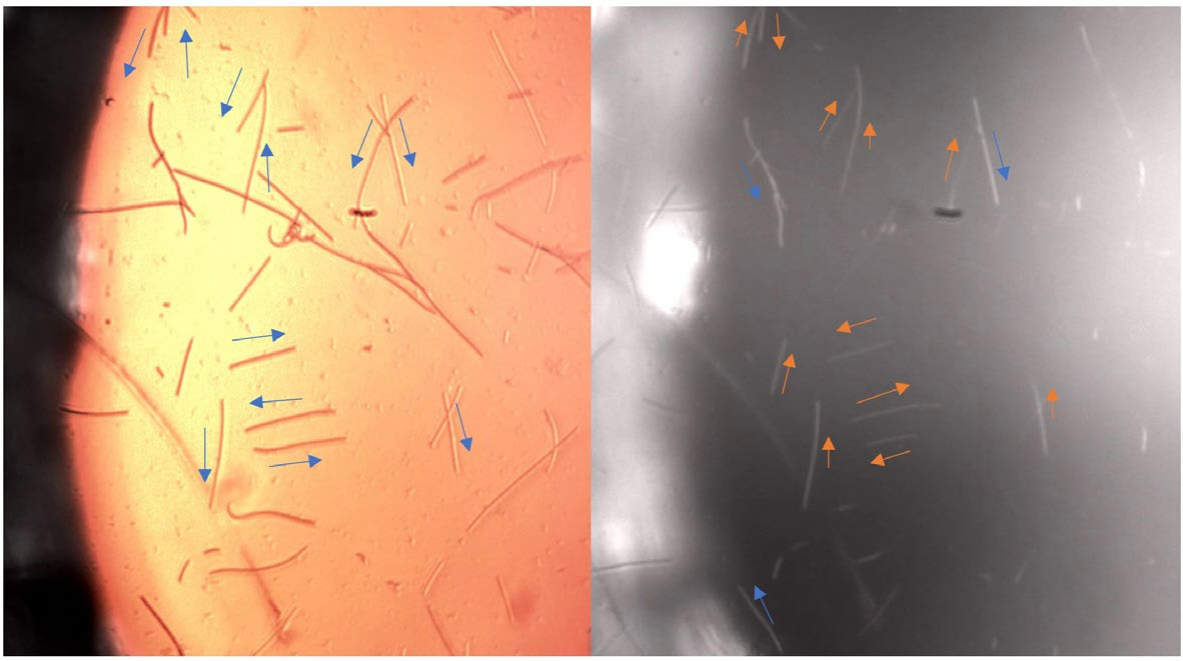
Direction change during red light to dark (i.e., infrared). Left and right panels show pictures of the same recording ca. 40 s before and just after the central red light was switched off. The movement directions as obtained from a comparison with a picture that was recorded 30 s after the printed one of selected individual filaments are indicated by arrows. The arrows are colored in blue and orange, orange means that the movement direction of the filament has changed after the light off. Table 3 shows further quantification.

To monitor the light–dark transition, filaments must experience a light gradient, which is either a spatial or a temporal gradient or both. We tested for a temporal gradient effect by switching the background red light on and off during the recording of the filament. A period length of 5 min of dark or light allowed for clear investigations. Figure 2 shows an example of how filaments change their movement direction after the light is off. The reversion during light on or light off was followed for 45 filaments from three recordings. 34 filaments changed their direction during light‐to‐dark transitions and 12 filaments changed their direction during dark to light transitions (Table 3). According to the Chi‐square test, the difference between both conditions was significant. Thus, the temporal light difference plays a significant role in the light trap mechanism. Whether or not a spatial light gradient along the filament induces changes in movement direction is difficult to investigate, because local microirradiation of moving objects is necessary, which is not established in our group.

**TABLE 3:** Filaments recorded under a 5 min light off/5 min light on cycle. About 45 filaments were traced manually and the direction change within the subsequent 30 sec was counted.

	Change of movement direction		No change
	During light‐to‐dark transition	During dark to light transition	
Number of filaments	34	12	11

### Movement between two positions

**FIGURE 3:**
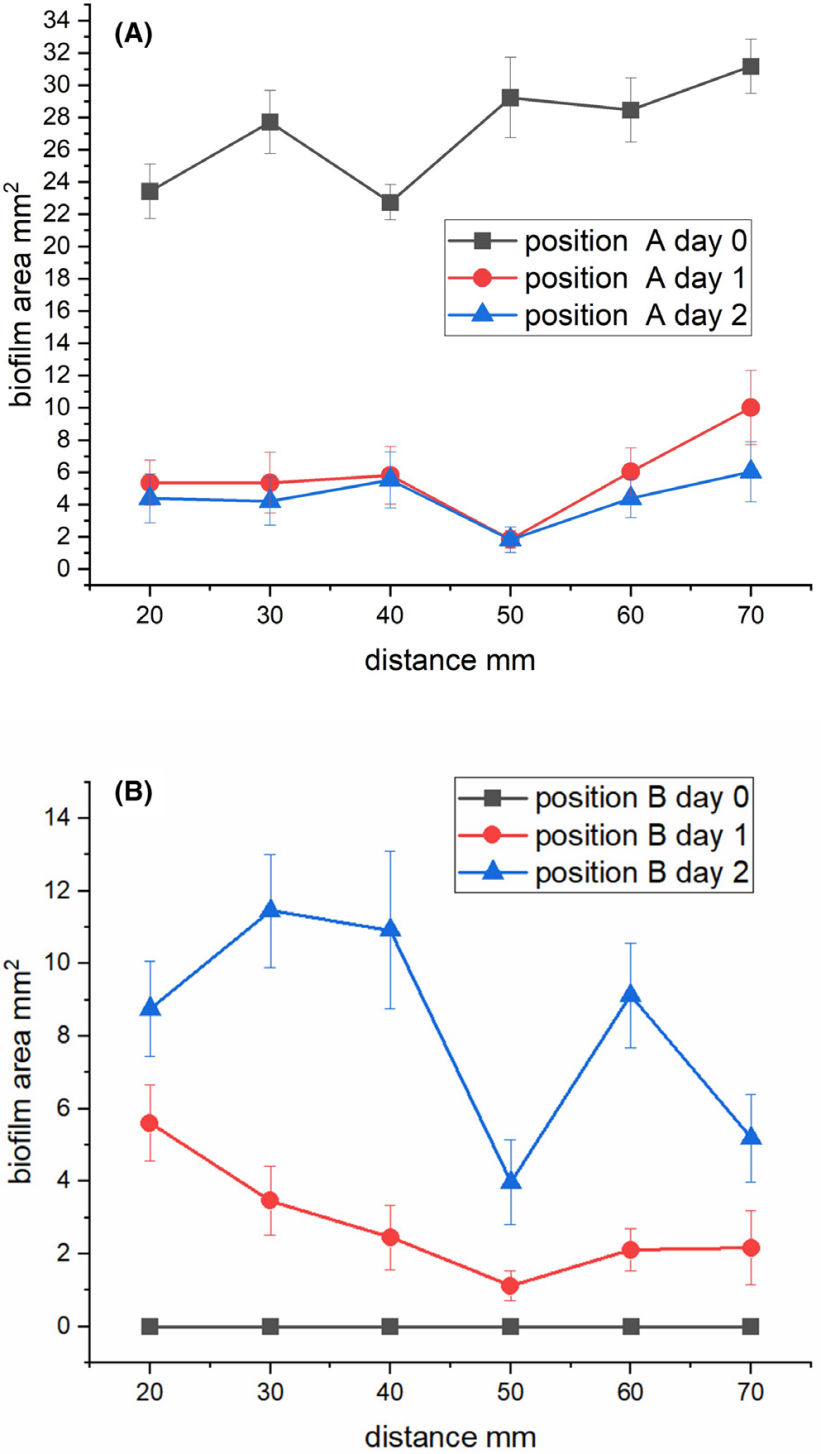
Movement over longer distance. *P. lacuna* filaments were subsequently irradiated at 2 positions with distances as given on the *x*‐axis. The biofilm areas at both positions (A and B) are indicated on the *y*‐axis.

To gain information about the long‐distance movement of *P. lacuna*, we induced migration from one spot to another at a defined distance. We began by irradiating a location 1 cm from the edge of a large Petri dish (9 cm diameter) containing a *P. lacuna* suspension (OD₇₈₀ = 0.3), using an LED (10 μmol m^−2^ s^−1^) from below for 4 days. At this position, designated as position A, the mean biofilm area ranged between 24 and 30 mm^2^. The filament solution outside the biofilm was then replaced by fresh medium so that all filaments observed thereafter must come from position A. Thereafter, the Petri dish was placed again on the LED so that the distance to position A was 2, 3, 4, 5, 6, or 7 cm. This is termed position B. The subsequent irradiation occurred for 1 or 2 days. A part of the filaments moved to the second position and formed a new biofilm. The biofilm areas at position A and B are plotted over the distances between A and B (Figure 3). After 1 day, the biofilm area at position B was between 1.1 and 5.6 mm^2^, and lay between 4 and 11 mm^2^ after 2 days. At the same time, the biofilm at position A decreased to between 4 and 10 mm^2^; the results for both days were similar. The maximum sum over point A and B reached 20 mm^2^, but was in most cases smaller, indicating that many but not all filaments reached the second position. We expected that there should be a negative correlation between the distance between A and B and the biofilm area at position B because fewer filaments should travel over a longer distance. This was found for day 1 for distances up to 5 cm, but with larger distances, the biofilm area increased again. At day 2 the biofilm area was around 10 mm^2^ for 2, 3, 4, and 6 cm and around 4 mm^2^ for 5 and 7 cm distance.

Given that the biofilm area is linear with the number of filaments, about 5% and 16% of the filaments traveled to the second light spot that is 7 cm apart from the first one within 1 or 2 days. At a movement speed of 130 μm min^−1^ (see above) and a straight movement the 7 cm distance could be reached within ca. 9 h. Thus, most if not all of the filaments change their movement direction on the way from position A to B, which also speaks for a random move model.

### Irradiation with two different wavelengths confirm adaptation through PixJ

**FIGURE 4:**
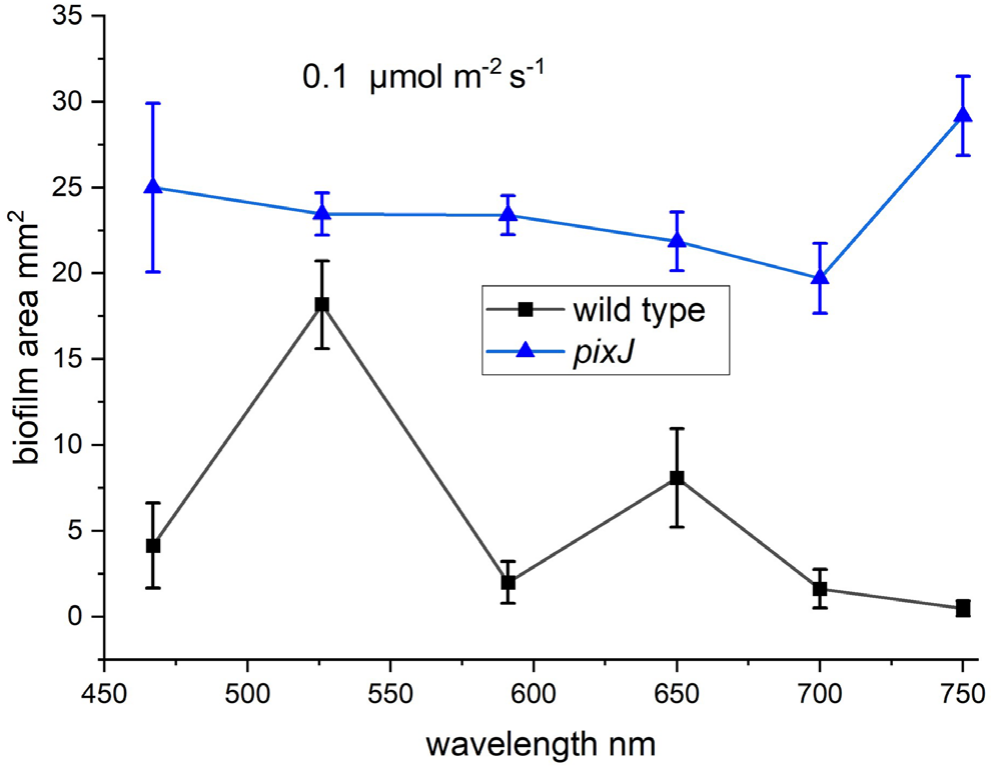
Action spectrum of *P. lacuna* wild‐type and mutant. Data are taken from.^5^ The original dataset was presented as intensity response curves; here only data at 0.1 μmol m^−2^ s^−1^ are shown. In the intensity ranges 0.01, 0.1, and 1 μmol m^−2^ s^−1^, the pixJ response was larger than that of wild‐type.

**FIGURE 5:**
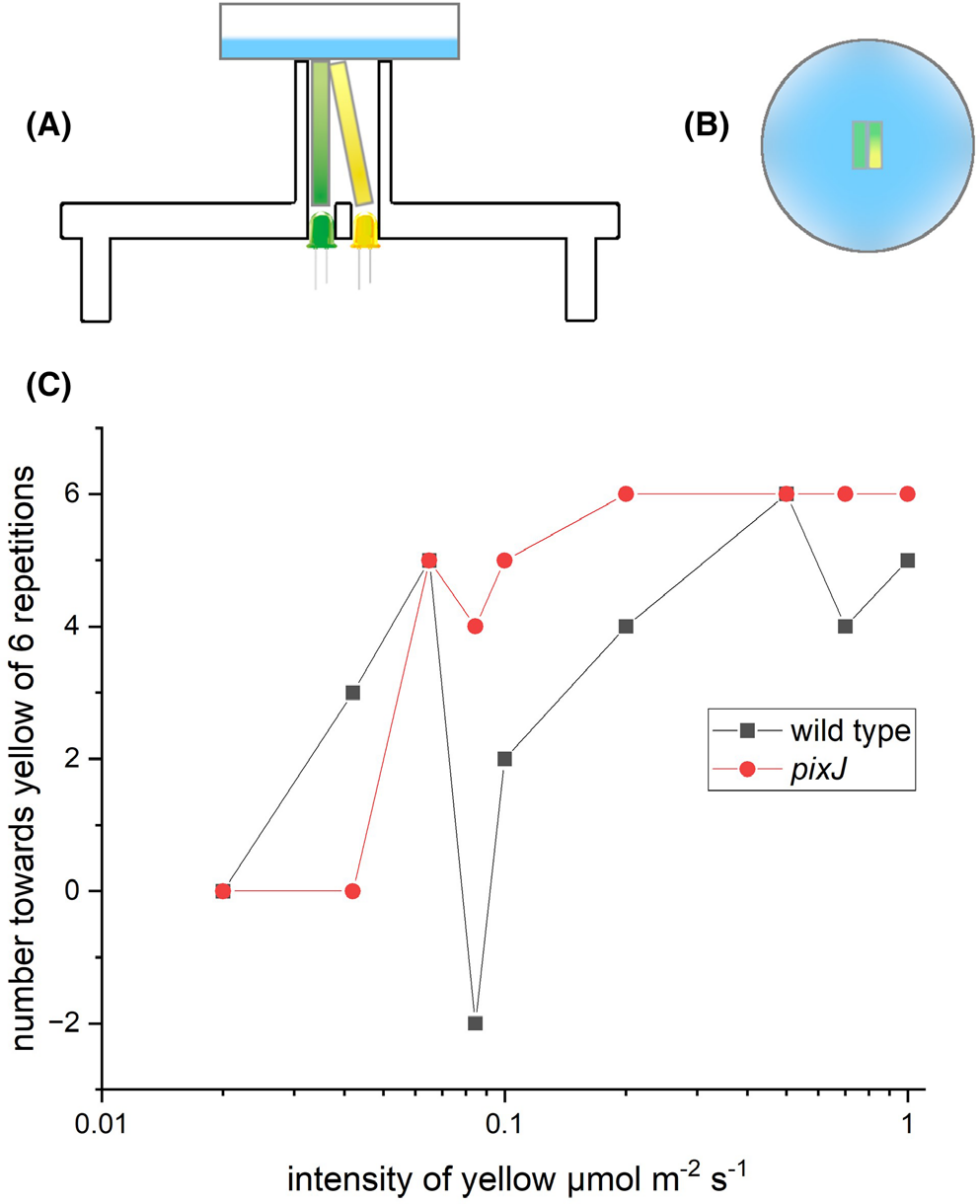
Dual irradiation of *P. lacuna* wild‐type and the pixJ mutant. Two rectangular plexiglas building blocks placed to each other irradiate *P. lacuna* from below. One end is yellow at variable intensity, the other is green with fix intensity of 1 μmol m^−2^ s^−1^. A and B, cartoons for arrangement of LED and plexiglas from the side (A) or above (B). (C) number of movements toward yellow plotted against the intensity of yellow. Each intensity was tested six times. The difference between wild‐type and pixJ at 0.085 μmol m^−2^ s^−1^ yellow is significant (Chi‐square test).

Previous experiments on the spectral effects of photophobotaxis have shown that red and blue light have a stronger influence on photophobotaxis than yellow light. In the pixJ mutant of *P. lacuna*, the photophobotactic response was stronger, and yellow light was equally effective as red and blue (see also Figure 4). This led to the assumption that PixJ inhibits photophobotaxis in the wild‐type, especially in the yellow range. We have tested this idea by using two different colors, green and yellow, and letting the filaments make a decision between both sides. Two rectangular light fields next to each other were illuminated with one or two LEDs, using plexiglas light guides so that a smooth transition from one field to the other is possible (see cartoon in Figure 5A,B). The experiment was performed with constant green light (1 μmol m^−2^ s^−1^) on both sides and additional yellow light of variable intensity on one side. The total light intensity on the yellow/green side is thus always higher than that on the other side. If yellow light acts inhibitory on photophobotaxis, filaments should decide for the other side with only green light. In the pixJ mutant yellow light should not lead to this effect.

For each yellow light intensity, six repetitions were performed. In each case, three different results could clearly be distinguished: (1) All filaments were on the side with only green; this event counted as −1; (2) all filaments were on the side with green and yellow; this event counted as +1; (3) filaments were equally distributed between both sides; this event counted as 0. The event values were added and plotted against the yellow light intensity (Figure 5C). Without yellow, the response was balanced between both sides. Most often, the additional yellow light drew the filaments on its side, in agreement with the overall light intensity on that side. For seven out of eight intensities, the values of wild‐type were smaller than or identical to the values of pixJ. In general, the mutant responded more sensitively to the additional yellow light, in accordance with previous results, although the differences were not significant (Mann–Whitney test). At 0.08 μmol m^−2^ s^−1^ the wild‐type had negative values; that is, in more cases, the filaments were found on the green side only. In the mutant, there was no such effect. The differences between mutant and wild‐type were significant (Chi‐square test). We can thus say that yellow light with 0.08 μmol m^−2^ s^−1^ reduces the sensitivity of the wild‐type filaments in such a way that they prefer green light alone and that this effect is mediated by PixJ.

## DISCUSSION

So far, photophobotaxis has been studied for only three cyanobacterial species. We have therefore undertaken a survey with 18 different cyanobacterial species. According to criteria outlined in detail in the results section, the responses indicated most often photophobotaxis and not photosynthetic growth. Thus, all seven single‐celled species, with the exception of a pilus‐free mutant of *Synechocystis* sp. PCC 6803, underwent photophobotaxis. Of the 11 filamentous species, only three did not show a response. To these belonged one of three Nostocales members (*Desmonostoc*) and one of each genera *Halomicronema* and *Toxifilum*. Vegetative filaments of Nostocales are thought to be immobile,^28^ and it was not unexpected that the *Desmonostoc* member did not show photophobotaxis. Also, species of the genus *Halomicronema* are reported to be immobile^29^ and it was not surprising to find that our *Halomicronema* strain does not undergo photophobotaxis. However, the responses of the Nostocales *Dolichospermum* sp. IPPAS B‐1213 and *Nodularia* sp. IPPAS B‐1529 were unexpected. Their light response could either indicate that vegetative filaments differentiated into motile hormogonia or that the responses were caused by photosynthetic growth. We indeed assume that photosynthetic growth is the reason for the spots observed here, because long irradiation times were required, because the patterns of the spots were irregular and because the spots remained stable upon transfer from LED to dark for several days. The *Toxifilum* sp. IPPAS B‐2086 results were also unexpected, but in the opposite way. This is the only species of the present survey that is expected to be motile but did not show a light response.

The involvement of type IV pili in gliding motility has been shown for several cyanobacteria and we assume that photophobotaxis is always also mediated by type IV pili, as shown earlier for *P*. *lacuna*.^5^ The aphotophobotactic character of the pilus‐free mutant used here supports this assumption. We assume that twitching motility and photophobotaxis are evolutionary basic phenomena of cyanobacteria and that motility was secondarily lost in a few species, leading to a loss in photophobotaxis. We also assume that the general mechanism for photophobotaxis is based on a random move and a change of direction upon light‐to‐dark transition, as has been observed in *P. lacuna* using infrared time‐lapse microscopy (Video S1).

For *O. lacuna* we also found that light stimulates aggregation– a light effect that might be initiated by photophobotaxis but could also hint at an aggregation process independent of light position and mediated by the extracellular polysaccharide matrix. Light stimulation of aggregation has been reported for a single‐celled cyanobacterium before^32^ and could play a role in other cyanobacteria.

If photophobotaxis is quasi‐universal among cyanobacteria, this would fit well with the proposed role of photosystems in light direction sensing, since photosystems are also universal in all cyanobacteria.

DCMU, an inhibitor of photosynthesis electron transport at the position of PS II inhibited photophobotaxis of single‐celled species and the three filamentous species at a concentration of 10 μM; only *P. lacuna* required higher concentrations of 100–1000 μM (Figure 1, Table 1,^5^). The answer to the earlier question of whether DCMU acts specifically in the inhibition of photophobotaxis is shifted toward “yes” in view of the lower concentrations in other species.

In further experiments with *P. lacuna*, we found that its movement speed was higher in darkness than in light. This effect makes sense, as filaments in the light do not need to change position to perform photosynthesis. While better conditions might exist nearby, long‐distance travel is unnecessary.

Continuous recording during light transitions (light on/off or off/on) showed that more filaments switched movement direction during the light off/on transition than during the reverse sequence (Figure 2). Most filaments recognized temporal changes in light intensity. The reversal of movement at the light–dark border, the basis of the “light trap,” is therefore likely based on temporal light differences, but spatial differences must also be relevant, because not all filaments changed their direction during light on/off. In filamentous species, signal transmission and communication throughout the entire filament are necessary for photophobotaxis. That filaments move toward stronger light contrasts with phototaxis of single‐celled species, where cells move away from brighter light.

The role of PixJ in phototaxis has been extensively studied in single‐celled species and in the photophobotaxis of *P. lacuna* using knockout mutagenesis.^10^ It has been suggested that PixJ regulates photophobotaxis sensitivity in *P. lacuna*, as the mutant exhibits heightened sensitivity to light of any color compared with the wild‐type.^5^ Here, we confirm the role of PixJ in photophobotaxis by studying movement in dual light fields: one green and one green/yellow with variable yellow intensity. In the pixJ mutant, yellow and green light always acted additively; as yellow light intensity increased, filaments were more frequently found on the yellow/green side. In contrast, wild‐type filaments exhibited a different response at a specific yellow light intensity, where they were more often found on the opposite side. This suggests that yellow light has not only an additive effect but also an antagonistic effect, which must be mediated by PixJ. This observation confirms the role of PixJ as a light‐induced downregulator of photophobotaxis sensitivity. The MCP domain in chemotaxis sensors plays a role in adapting to chemical concentration changes.^33^ The homologous domain in PixJ may also be involved in adaptation, though the mechanism must differ from chemotaxis, where MCP is part of the sensor protein.

We also made the observation that significant fractions of filaments *P. lacuna* moved from one light spot to a 7 cm distant light spot within 1 or 2 days. With a migration speed of 130 μm min^−1^ a direct move would take only 9 h, so the result provides further evidence for random movement. Based on these observations, we propose that during the night, filaments spread out over several centimeters, and on the next day, the major fraction finds a place in the light to make photosynthesis. If not, there is at least a chance for a small fraction to build a new culture.

In conclusion, we show that photophobotaxis is a light effect of a vast majority of cyanobacteria that is probably coupled to PS II as a sensor for light intensity changes. Because the effect can easily be studied for single‐celled and filamentous species, we propose that investigations of photophobotaxis will increase.

## FUNDING INFORMATION

This work was funded by the KIT and the infrastructural support from the Ministry of Science and Higher Education of the Russian Federation (theme #122042700045‐3).

## Supporting information


**Video S1.** Time‐lapse recording of *P. lacuna* filaments between red LED and infrared. Filaments move in random directions but do not leave the red light.

## Data Availability

The data that support the findings of this study are available from the corresponding author upon reasonable request.

## References

[1] Strunecky O , Ivanova AP , Mares J . An updated classification of cyanobacterial orders and families based on phylogenomic and polyphasic analysis. J Phycol. 2023;59:12‐51. 36443823 10.1111/jpy.13304

[2] Bhaya D , Bianco NR , Bryant D , Grossman A . Type IV pilus biogenesis and motility in the cyanobacterium Synechocystis sp. PCC6803. Mol Microbiol. 2000;37:941‐951. 10972813 10.1046/j.1365-2958.2000.02068.x

[3] Lamparter T , Babian J , Frohlich K , et al. The involvement of type IV pili and the phytochrome CphA in gliding motility, lateral motility and photophobotaxis of the cyanobacterium Phormidium lacuna. PLoS One. 2022;17:e0249509. 35085243 10.1371/journal.pone.0249509 PMC8794177

[4] Fiedler B , Börner T , Wilde A . Phototaxis in the cyanobacterium Synechocystis sp. PCC 6803: role of different photoreceptors. Photochem Photobiol. 2005;81:1481‐1488. 16354116 10.1562/2005-06-28-RA-592

[5] Schwabenland E , Jelen CJ , Weber N , Lamparter T . Photophobotaxis in the filamentous cyanobacterium Phormidium lacuna: mechanisms and implications for photosynthesis‐based light direction sensing. Photochem Photobiol. 2024;100:1290‐1309. 38269403 10.1111/php.13908

[6] Choi JS , Chung YH , Moon YJ , et al. Photomovement of the gliding cyanobacterium Synechocystis sp. PCC 6803. Photochem Photobiol. 1999;70:95‐102. 10420848 10.1562/0031-8655(1999)070<0095:potgcs>2.3.co;2

[7] Nakane D , Enomoto G , Baehre H , Hirose Y , Wilde A , Nishizaka T . Thermosynechococcus switches the direction of phototaxis by a c‐di‐GMP‐dependent process with high spatial resolution. Elife. 2022;11:30. 10.7554/eLife.73405 PMC9090330 35535498

[8] Lamparter T . Photosystems and photoreceptors in cyanobacterial phototaxis and photophobotaxis. FEBS Lett. 2024;598:1899‐1908. 38946046 10.1002/1873-3468.14968

[9] Nultsch W . Der einfluss des lichtes auf die bewegung der cyanophyceen. 3. Photophobotaxis von phormidium‐uncinatum. Planta. 1962;58:647‐663.

[10] Yoshihara S , Katayama M , Geng X , Ikeuchi M . Cyanobacterial phytochrome‐like PixJ1 holoprotein shows novel reversible photoconversion between blue‐ and green‐absorbing forms. Plant Cell Physiol. 2004;45:1729‐1737. 15653792 10.1093/pcp/pch214

[11] Narikawa R , Fukushima Y , Ishizuka T , Itoh S , Ikeuchi M . A novel photoactive GAF domain of cyanobacteriochrome AnPixJ that shows reversible green/red photoconversion. J Mol Biol. 2008;380:844‐855. 18571200 10.1016/j.jmb.2008.05.035

[12] Fiedler B , Broc D , Schubert H , Rediger A , Börner T , Wilde A . Involvement of cyanobacterial phytochromes in growth under different light qualities and quantities. Photochem Photobiol. 2004;79:551‐555. 15291308 10.1562/rn-013r.1

[13] Allen MM , Stanier RY . Selective ISOLATION of blue‐green algae form water and soil. J Gen Microbiol. 1968;51:203. 5652096 10.1099/00221287-51-2-203

[14] Zarrouk C . Contribution a l'etude d'une Cyanophycee. Influence de Divers Facteurs Physiques et Chimiques sur la croissance et la photosynthese de Spirulina mixima. *Thesis*. University of Paris, France; 1966.

[15] Guillard RR , Ryther JH . Studies of marine planktonic diatoms. I. Cyclotella nana Hustedt, and Detonula confervacea (cleve) gran. Can J Microbiol. 1962;8:229‐239. 13902807 10.1139/m62-029

[16] Vladimirova MGB , Zholdakov IA , Epifanova OO , Markelova AG , Maslova IP , Kuptsova ES . Growth media. In: Semenenko VE , ed. IPPAS—Collection of Microalgae of Timiryazev Institute of Plant Physiology, Acad. Sci. USSR. In Katalog Kul'tur Mikrovodoroslei v Kollektsiyakh SSSR (Catalogue of Microalgal Cultures in the Collections of USSR). Russian Academy Science; 1991:8‐61.

[17] Samylina OS , Sinetova MA , Kupriyanova EV , et al. Ecology and biogeography of the ‘marine Geitlerinema’ cluster and a description of Sodalinema orleanskyi sp. nov., Sodalinema gerasimenkoae sp. nov., Sodalinema stali sp. nov. and Baaleninema simplex gen. Et sp. nov. (Oscillatoriales, cyanobacteria). FEMS Microbiol Ecol. 2021;97:1‐25. 10.1093/femsec/fiab104 34254131

[18] Stanier RY , Kunisawa R , Mandel M , Cohen‐Bazire G . Purification and properties of unicellular blue‐green algae (order Chroococcales). Bacteriol Rev. 1971;35:171‐205. 4998365 10.1128/br.35.2.171-205.1971 PMC378380

[19] Sarsekeyeva FK , Usserbaeva AA , Zayadan BK , et al. Isolation and characterization of a new cyanobacterial strain with a unique fatty acid composition. Adv Microbiol. 2014;4:1033‐1043.

[20] Bozieva AM , Khasimov MK , Voloshin RA , et al. New cyanobacterial strains for biohydrogen production. Int J Hydrogen Energy. 2023;48:7569‐7581.

[21] Bürgi H . Long‐term development of blue‐green‐algae in lake Lucerne with special reference to Oscillatoria (Planktothrix) rubescens. Schweizerische Zeitschrift Fur Hydrologie‐Swiss J Hydrol. 1987;49:375‐377.

[22] Nies F , Worner S , Wunsch N , et al. Characterization of *Phormidium lacuna* strains from the North Sea and the Mediterranean Sea for biotechnological applications. Process Biochem. 2017;59:194‐206.

[23] Bilova T , Golushko N , Frolova N , et al. Strain‐specific features of primary metabolome characteristic for extremotolerant/extremophilic cyanobacteria under long‐term storage. Int J Mol Sci. 2025;26:2201. 40076823 10.3390/ijms26052201 PMC11900582

[24] Bataeva YV , Satkalieva MS , Antonova SV , et al. Study of antioxidant activity and composition of cyanobacteria metabolites by TLC, HPTLC, and HPLC for the search of environmentally safe cleaning agents. Russ J Gen Chem. 2018;88:2898‐2902.

[25] Sinetova MA , Bolatkhan K , Sidorov RA , et al. Polyphasic characterization of the thermotolerant cyanobacterium Desertifilum sp. strain IPPAS B‐1220. FEMS Microbiol Lett. 2017;364:1‐9. 10.1093/femsle/fnx027 28130365

[26] Trautmann D , Voss B , Wilde A , Al‐Babili S , Hess WR . Microevolution in cyanobacteria: re‐sequencing a motile substrain of Synechocystis sp. PCC 6803. DNA Res. 2012;19:435‐448. 23069868 10.1093/dnares/dss024 PMC3514855

[27] Qian M , Han X , Liu J , Xu P , Tao F . Genomic insights on the carbon‐negative workhorse: systematical comparative genomic analysis on 56 Synechococcus strains. Bioengineering. 2023;10:1329. 38002453 10.3390/bioengineering10111329 PMC10669429

[28] Risser DD . Hormogonium development and motility in filamentous cyanobacteria. Appl Environ Microbiol. 2023;89:e0039223. 37199640 10.1128/aem.00392-23 PMC10304961

[29] Ruocco N , Mutalipassi M , Pollio A , Costantini S , Costantini M , Zupo V . First evidence of Halomicronema metazoicum (cyanobacteria) free‐living on Posidonia oceanica leaves. PLoS One. 2018;13:e0204954. 30273387 10.1371/journal.pone.0204954 PMC6166977

[30] Zimba PV , Huang I‐S , Foley JE , Linton EW . Identification of a new‐to‐science cyanobacterium, Toxifilum mysidocida gen. Nov. & sp. nov. (cyanobacteria, Cyanophyceae). J Phycol. 2017;53:188‐197. 27809340 10.1111/jpy.12490

[31] Kupriyanova EV , Cho SM , Park Y‐I , Pronina NA , Los DA . The complete genome of a cyanobacterium from a soda lake reveals the presence of the components of CO_2_‐concentrating mechanism. Photosynth Res. 2016;130:151‐165. 26908147 10.1007/s11120-016-0235-0

[32] Enomoto G , Ni W , Narikawa R , Ikeuchi M . Three cyanobacteriochromes work together to form a light color‐sensitive input system for c‐di‐GMP signaling of cell aggregation. Proc Natl Acad Sci U S A. 2015;112:8082‐8087. 26080423 10.1073/pnas.1504228112 PMC4491779

[33] Gegner JA , Graham DR , Roth AF , Dahlquist FW . Assembly of an MCP receptor, CheW, and kinase CheA complex in the bacterial chemotaxis signal transduction pathway. Cell. 1992;70:975‐982. 1326408 10.1016/0092-8674(92)90247-a

